# MKP-5 Relieves Lipotoxicity-Induced Islet β-Cell Dysfunction and Apoptosis via Regulation of Autophagy

**DOI:** 10.3390/ijms21197161

**Published:** 2020-09-28

**Authors:** Tongjian Zhao, Jie Ma, Lulu Li, Wenjing Teng, Yafei Tian, Yongjun Ma, Wei Wang, Weiqun Yan, Ping Jiao

**Affiliations:** School of Pharmaceutical Sciences, Jilin University, 1266 Fujin Road, Changchun 130021, China; zhaotj19@mails.jlu.edu.cn (T.Z.); ma_jie@jlu.edu.cn (J.M.); lill14@mails.jlu.edu.cn (L.L.); tengwj18@mails.jlu.edu.cn (W.T.); tianyf19@mails.jlu.edu.cn (Y.T.); mayj19@mails.jlu.edu.cn (Y.M.); wangwei970822@jlu.edu.cn (W.W.)

**Keywords:** MKP-5, islet cells, lipotoxicity, autophagy

## Abstract

Mitogen-activated protein kinase phosphatase-5 (MKP-5) is a regulator of extracellular signaling that is known to regulate lipid metabolism. In this study, we found that obesity caused by a high-fat diet (HFD) decreased the expression of MKP-5 in the pancreas and primary islet cells derived from mice. Then, we further investigated the role of MKP-5 in the protection of islet cells from lipotoxicity by modulating MKP-5 expression. As a critical inducer of lipotoxicity, palmitic acid (PA) was used to treat islet β-cells. We found that MKP-5 overexpression restored PA-mediated autophagy inhibition in Rin-m5f cells and protected these cells from PA-induced apoptosis and dysfunction. Consistently, a lack of MKP-5 aggravated the adverse effects of lipotoxicity. Islet cells from HFD-fed mice were infected using recombinant adenovirus expressing MKP-5 (Ad-MKP-5), and we found that Ad-MKP-5 was able to alleviate HFD-induced apoptotic protein activation and relieve the HFD-mediated inhibition of functional proteins. Notably, HFD-mediated impairments in autophagic flux were restored by Ad-MKP-5 transduction. Furthermore, the autophagy inhibitor 3-methyladenine (3-MA) was used to treat Rin-m5f cells, confirming that the MKP-5 overexpression suppressed apoptosis, dysfunction, inflammatory response, and oxidative stress induced by PA via improving autophagic signaling. Lastly, employing c-Jun amino-terminal kinas (JNK), P38, or extracellular-regulated kinase (ERK) inhibitors, we established that the JNK and P38 MAPK pathways were involved in the MKP-5-mediated apoptosis, dysfunction, and autophagic inhibition observed in islet β cells in response to lipotoxicity.

## 1. Introduction

Type 2 diabetes mellitus (T2DM) is highly prevalent worldwide, with an incidence of 8.3% that is predicted to rise as high as 10.1% by 2035 [1,2]. This disease is complicated and characterized by hyperglycemia and impaired insulin secretion by islet β-cells [3,4]. More than 90% of T2DM patients are obese or overweight. Elevated levels of free fatty acids (FFAs) released from the enlarged and stressed adipose tissue are commonly detected in these obese individuals [5,6]. Palmitic acid (PA), as a major FFA component, is a primary cause of lipotoxicity in islet β-cells, leading to their dysfunction and apoptosis via endoplasmic reticulum stress (ERS) and the inflammatory response [7,8].

Autophagy is an intracellular process wherein cellular metabolic homeostasis is maintained via the recycling of specific proteins and organelles. Autophagic flux can be blocked by PA owing to its ability to impair the conversion of lysosomes to autophagosomes, resulting in the apoptotic death of islet β cell lines and human pancreatic islets [9,10]. In contrast, other studies suggest that FFAs can induce autophagy [11,12]. Recently, autophagy has also been reported to mediate ERS, inflammation, and oxidative stress [13,14,15], although the exact mechanisms underlying these effects remain to be fully elucidated. As such, the molecular mechanisms whereby FFAs influence autophagy in order to drive islet β-cell dysfunction and death require further study. 

Mitogen-activated protein kinases (MAPKs) are vital mediators of cellular signaling associated with the pathogenesis of obesity induced-insulin resistance and T2DM [16], with c-Jun amino-terminal kinase (JNK), P38 protein kinase, and extracellular-regulated kinase (ERK) being the principle relevant MAPKs [17]. There is growing evidence that MAPK signaling, as a critical regulator of apoptosis and cellular dysfunction, is tightly associated with ERS, oxidative stress, autophagy, and the inflammatory response [18,19,20,21]. MAPK activation is determined based on co-regulation by a range of specific protein kinases and phosphatases, and the precise mechanisms negatively regulating MAPK activation in the context of obesity-related T2DM remain to be determined.

As the upstream regulators of MAPKs, mitogen-activated protein kinase phosphatases (MKPs), also known as dual specific phosphatases (DUSPs), can negatively regulate MAPK phosphatases via direct dephosphorylation [22]. Many researchers have identified a pivotal role for these MKPs as regulators of T2DM. For example, the phosphatase activity of MKP-3 is essential for forkhead box O1(FOXO1) nuclear translocation and the promotion of gluconeogenesis [23]. Similarly, MKP-4 has a protective effect against the development of insulin resistance and is known to inactivate crucial mediators of stress-induced insulin resistance such as ERK and JNK [24]. Conversely, another study has suggested that MKP-4 negatively regulates insulin signaling, potentially thereby contributing to the pathogenesis of insulin resistance [25]. In addition, increasing evidence indicates that MKPs can regulate autophagy in the context of MKP-1 knockdown via increasing both basal and rapamycin-induced autophagic flux [26]. Recently, one study has indicated that MKP-5 limits reactive oxygen species (ROS) production and can prevent lipopolysaccharide (LPS) -induced vascular injury in mice [27]. MKP-5 is also known to play a potential role in obesity-related metabolic disease, alleviating inflammation in certain cell types via the P38 and JNK pathways [28,29]. However, little is known regarding whether MKP-5 plays a role in the obesity-related apoptosis and autophagy of islet β-cells. Therefore, in the present study, we assessed this question, determining that MKP-5 suppresses obesity-induced apoptosis, ERS, oxidative stress, inflammation, and glucose-stimulated insulin secretion (GSIS) in islet cells via inducing autophagy. Furthermore, we found that MKP-5 protects islet cells from lipotoxicity-induced apoptosis and dysfunction by promoting autophagy flux mainly via the negative regulation of the P38 and JNK MAPK pathways.

## 2. Results

### 2.1. Overexpression of MKP-5 Reduces PA-Induced Growth Inhibition and Apoptosis in Rin-m5f Cells

**Figure 1:**
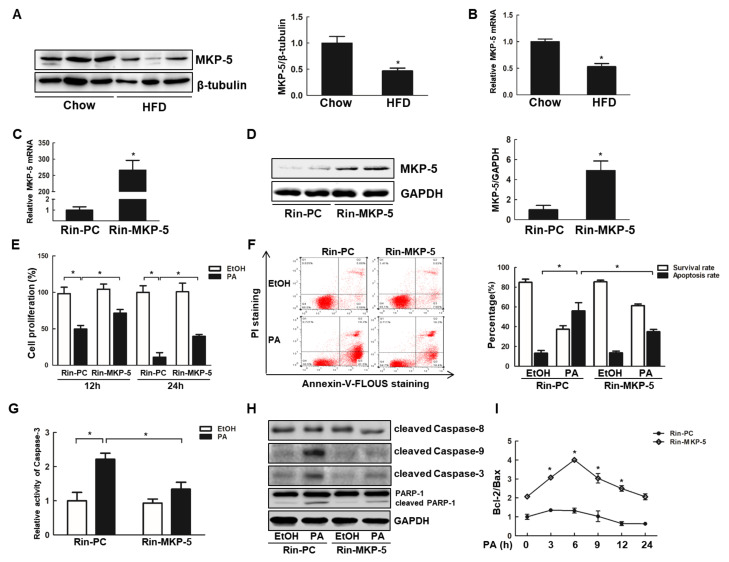
Overexpression of mitogen-activated protein kinase phosphatase-5 (MKP-5) relieves the apoptosis of Rin-m5f cells upon palmitic acid (PA) exposure. (**A**) Representative pancreatic MKP-5 expression in mice fed high-fat diet (HFD) or chow as measured by Western blotting (male mice; *n* = 7 per group, left). Levels of MKP-5 were quantified and normalized to β-tubulin in these same mice (right). (**B**) The expression of MKP-5 in islet cells from mice fed HFD or chow was measured by real-time PCR (male mice; *n* = 7 per group). (**C**,**D**) The Rin-PC and Rin-MKP-5 cell lines were constructed, and the level of MKP-5 was measured by real-time PCR (**C**) and Western blotting. ((**D**), Left) Levels of MKP-5 were quantified and normalized to glyceraldehyde-3-phosphate dehydrogenase (GAPDH) ((**D**), Right). (**E**) The cell proliferation of Rin-PC and Rin-MKP-5 cells treated with PA for 12 and 24 h was measured via 3-(4,5-dimethylthiazol-2-yl)-5-(3-carboxymethoxyphenyl)-2-(4-sulfophenyl)-2H-tetrazolium, inner salt (MTS) assay. (**F**) The apoptotic ratios of the cells following a 24 h palmitic acid (PA) treatment were quantified based upon Annexin V and propidium iodide (PI) staining results. (**G**) Caspase-3 activity was measured using Ac-DEVD-PNA in Rin-m5f cells exposed to PA for 24 h. (**H**) Levels of cleaved Caspase-9, cleaved Caspase-8, cleaved Caspase-3, and cleaved poly (ADP-ribose) polymerase-1 (PARP-1) in Rin-PC and Rin-MKP-5 cells treated with PA for 9 h were measured via Western blotting. (**I**) The relative levels of B-cell lymphoma-2 (Bcl-2)/ Bcl-2 associated X protein (Bax) were measured by real-time PCR in Rin-m5f cells treated with PA for the indicated times. *, *p* < 0.05, Rin-PC vs. Rin-MKP-5; EtOH vs. PA; HFD vs. chow.

To determine the role of MKP-5 in lipotoxicity-related islet cell apoptosis and dysfunction, we assessed the levels of MKP-5 in the pancreas and islet cells isolated from mice fed with a high-fat diet (HFD). The expression of MKP-5 in the pancreas (Figure 1A) and islet cells (Figure 1B) was substantially lower in HFD mice relative to chow-fed controls. To explore the implications of this finding, an MKP-5 overexpressing cell line (Rin-MKP-5) and a control cell line (Rin-PC) were constructed (Figure 1C,D). In these cells, we found that the survival rate of Rin-PC cells exposed to PA was reduced from ≈50% at 12 h to ≈10% at 24 h following treatment (*p* < 0.05). In contrast, the survival rate of Rin-MKP-5 cells treated with PA was significantly higher than that of Rin-PC cells at the corresponding time points (Figure 1E). In addition, Annexin V-Fluorescein (FLOUS) and propidium iodide (PI) double staining was employed to further analyze the effects of MKP-5 expression on PA-induced apoptosis in Rin-m5f cells. Consistent with the above results, the percentage of apoptotic cells quantified by flow cytometry in the Rin-MKP-5 group was markedly reduced relative to in the Rin-PC group, decreasing from 56.05 ± 8.26% to 34.98 ± 2.41% after 24 h PA treatment (Figure 1F).

Caspase-3 is a key terminal enzyme in the apoptotic process. The activity of Caspase-3 in Rin-PC cells was markedly increased nearly 2-fold in the PA-treated group relative to EtOH-treated controls. MKP-5 overexpression decreased this Caspase-3 activation by almost 70% in PA-treated Rim-m5f cells (Figure 1G), further demonstrating that MKP-5 participated in the PA-induced apoptosis of Rin-m5f cells. In addition, the expression of apoptotic proteins, such as cleaved Caspase-9, cleaved Caspase-3, and cleaved PARP-1 were all upregulated following a 9 h PA treatment in Rin-m5f cells, whereas the overexpression of MKP-5 decreased the PA-dependent induction of these proteins (Figure 1H). We observed no effect of PA on Caspase-8 activation in any of these groups. Moreover, the relative expression of Bcl-2/Bax, which are proteins upstream of the mitochondrial pathway, were significantly elevated in Rin-MKP-5 cells group as compared with Rin-PC cells group following PA treatment. The relative levels of Bcl-2/Bax peaked at 6 h and then gradually decreased in Rin-MKP-5 cells (Figure 1I). Together, these results suggested that MKP-5 might be a key regulator inhibiting PA-induced apoptosis through the mitochondrial pathway and not through the death receptor pathway in Rin-m5f cells.

### 2.2. MKP-5 Regulates PA-Induced Inflammation, Oxidative Stress, and Impairment of Insulin Secretion in Rin-m5f Cells

**Figure 2:**
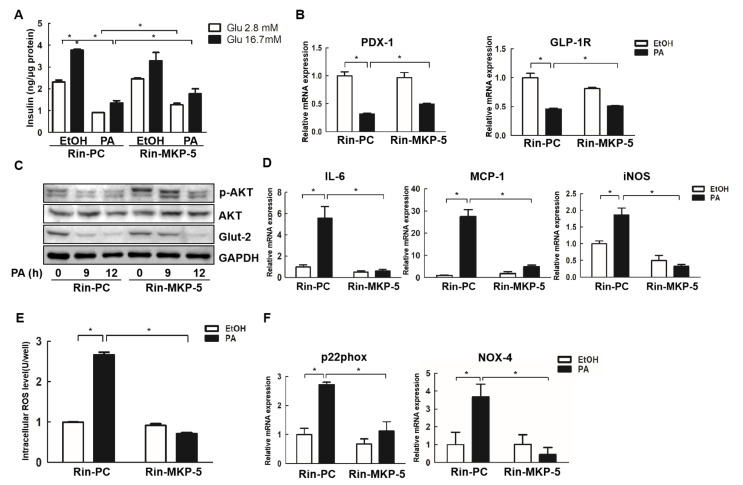
MKP-5 decreases inflammation, oxidative stress, and preserves the function of Rin-m5f cells in response to PA treatment. (**A**) Glucose-stimulated insulin secretion (GSIS) responses were determined by ELISA in Rin-MKP-5 and Rin-PC cells treated with PA for 24 h. (**B**) The relative mRNA expression of pancreatic duodenal homeobox factor-1 (PDX-1) and glucagon-like peptide-1 receptor (GLP-1R) were measured by real-time PCR in Rin-m5f cells upon 12 h PA treatment. (**C**) The protein levels of p-AKT, AKT, and Glut-2 were determined in Rin-PC and Rin-MKP-5 cells upon PA stimulation for 9 h and 12 h. (**D**) Expression of the inflammation-related genes inducible nitric oxide synthase (iNOS), interleukin-6 (IL-6), and monocyte chemotactic protein-1 (MCP-1) were detected by real-time PCR in Rin-m5f cells upon PA exposure for 12 h. (**E**) Levels of PA-induced intracellular reactive oxygen species (ROS) in Rin-PC and Rin-MKP-5 cells were determined using a fluorescence microplate reader by measuring DCFH-DA-fluorescence. (**F**) The expression of the ROS-related genes p22phox and NADPH oxidase-4 (Nox-4) was analyzed by real-time PCR. *, *p* < 0.05, EtOH vs. PA; Rin-PC vs. Rin-MKP-5; Glu 2.8 mM vs. Glu 16.7 mM.

We next investigated the relationship between MKP-5 and the function of Rin-m5f cells. Different concentrations of glucose were used to stimulate insulin secretion in Rin-PC and Rin-MKP-5 cells. We found that high concentrations of glucose (16.7 mM) enhanced the release of insulin from 2.32 ± 0.09 ng/μg to 3.78 ± 0.06 ng/μg relative to basal concentration (2.8 mM) in Rin-PC cells. Upon PA treatment, the insulin secretion of Rin-m5f cells was decreased more than 50% upon exposure to both 2.8 mM and 16.7 mM glucose (*p* < 0.05), indicating that PA impaired glucose-stimulated insulin secretion (GSIS) in these Rin-m5f cells. However, MKP-5 overexpression increased the insulin secretion from 0.90 ± 0.01 to 1.27 ± 0.07 ng/μg upon 2.8 mM glucose stimulation, and from 1.36 ± 0.10 to 1.78 ± 0.22 ng/μg upon 16.7 mM glucose stimulation in PA-treated Rin-MKP-5 cells relative to Rin-PC cells treated with PA (*p* < 0.05) (Figure 2A). Pancreatic duodenal homeobox factor-1 (PDX-1) is a transcription factor necessary for pancreatic development, and it is also known as a marker of islet β-cell functionality. The glucagon-like peptide-1 receptor (GLP-1R) is a receptor found on islet β-cells, which is involved in the control of blood sugar levels by enhancing insulin secretion. The relative levels of PDX-1 and GLP-1R were significantly decreased in PA-treated Rin-m5f cells, whereas the overexpression of MKP-5 could relieve the inhibition of PDX-1 and GLP-1R in these cells after they had been treated with PA for 12 h (*p* < 0.05) (Figure 2B). Glucose transporter-2 (Glut-2) is a transmembrane carrier protein that facilitates glucose transport across cell membranes and enters the cell. The phosphorylation of AKT also triggers the insulin secretion. We found that the expression of p-AKT was downregulated in Rin-PC cells exposed to PA in a time-dependent manner, but MKP-5 overexpression substantially increased the levels of p-AKT in Rin-MKP-5 cells relative to Rin-PC cells upon PA treatment at both 0 and 9 h post-treatment (Figure 2C). In addition, MKP-5 overexpression relieved the inhibition of Glut-2 in Rin-m5f cells upon PA treatment for 9 h (Figure 2C).

Inflammatory processes have also been implicated as mediators of defective islet β-cell functionality upon PA treatment [30]. PA also markedly increased the relative expression of inflammation-related factors, including interleukin-6 (IL-6), monocyte chemotactic protein-1 (MCP-1), and inducible nitric oxide synthase (iNOS) in Rin-PC cells, whereas this effect was abolished upon MKP-5 overexpression (Figure 2D).

Higher levels of ROS have been found in the islets of T2DM patients, and ROS weakens insulin secretion in this context [31]. To assess whether MKP-5 overexpression could inhibit PA-induced intracellular ROS, Rin-m5f cells were exposed to PA for 24 h. We found that PA increased ROS levels roughly 3-fold in Rin-PC cells (*p* < 0.05). However, the overexpression of MKP-5 nearly completely neutralized this PA-mediated ROS induction (*p* < 0.05) (Figure 2E). In addition, MKP-5 overexpression markedly decreased the relative expression of the NAD(P)H oxidase enzyme-related genes, Nox-4 and p22phox, which had been elevated upon PA treatment, indicating that MKP-5 may reduce PA-induced ROS production by inhibiting the expression of Nox-4 and p22phox (Figure 2F). Collectively, these results suggested that MKP-5 is capable of protecting Rin-m5f cells from PA-induced dysfunction, inflammation, and oxidative stress.

### 2.3. Knockdown of MKP-5 Exacerbates Lipotoxicity-Induced Apoptosis and Dysfunction in Rin-m5f Cells

**Figure 3:**
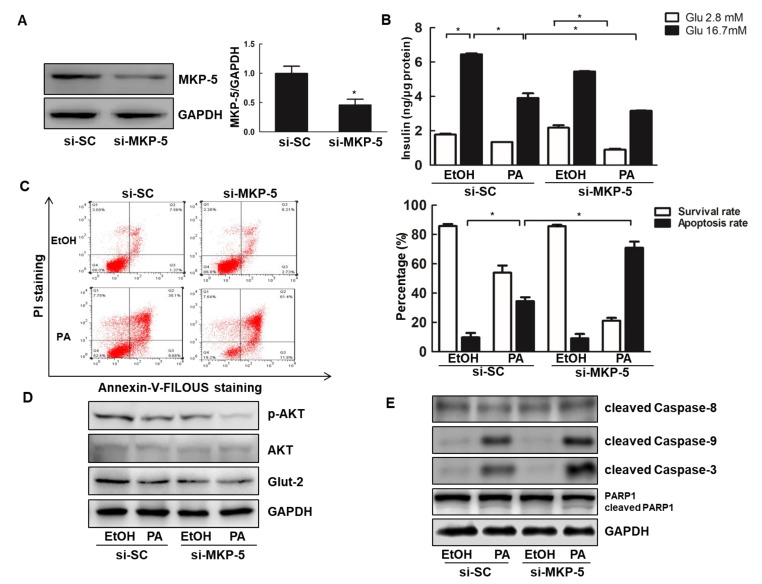
Knockdown of MKP-5 aggravates Rin-m5f cell apoptosis and dysfunction upon PA treatment. (**A**) The expression of MKP-5 was assessed via Western blotting in Rin-m5f cells transfected with si-RNA against MKP-5 (si-MKP-5) or scramble siRNA control (si-SC) for 72h. (**B**) GSIS responses were determined in Rin-m5f cells transfected with si-MKP-5 upon PA treatment for 16 h. (**C**) The ratios of apoptotic Rin-m5f cells treated with PA for 16 h were quantified by flow cytometry using an Annexin V-FLOUS and propidium iodide (PI) double staining kit. (**D**,**E**) Rin-m5f cells transfected with si-SC or si-MKP-5 were exposed to PA for 9 h. The expression of AKT, p-AKT, and Glut-2 was determined via Western blotting (**C**). The levels of AKT, p-AKT, and Glut-2 were analyzed by Western blotting. *, *p* < 0.05, si-SC vs. si-MKP-5; EtOH vs. PA; GLU 2.8 mM vs. GLU 16.7 mM.

To further explore the connection between MKP-5 and PA-induced apoptosis of islet cells, we next inhibited the expression of MKP-5 using a specific small interfering RNA (siRNA) in Rin-m5f cells. After confirming that MKP-5 expression was effectively reduced (Figure 3A), insulin secretion was quantified by ELISA. PA reduced insulin levels produced in response to both glucose concentrations in Rin-m5f cells transfected with siRNAs, with the effect being more pronounced for cells transfected with si-MKP-5 (Figure 3B). Furthermore, the rates of apoptosis in these Rin-m5f cells were quantified by flow cytometry using an Annexin V-FLOUS and propidium iodide (PI) double staining kit. The percentage of apoptotic cells in the si-MKP-5 group was markedly increased as compared to the cells in the si-SC group from (34.58 ± 2.67% vs. 70.97 ± 4.22%) at 16 h post-PA-treatment, indicating that the inhibition of MKP-5 may aggravate PA-related apoptosis (Figure 3C). Moreover, MKP-5 knockdown reduced the protein expression of p-AKT and Glut-2 in Rin-MKP-5 cells relative to Rin-PC cells, regardless of PA treatment status (Figure 3D). Consistent with this, the levels of apoptotic proteins including cleaved Caspase-9, cleaved Caspase-3, and cleaved PARP-1 were all increased in Rin-m5f cells upon PA stimulation for 9 h, with no apparent effect on cleaved Caspase-8. Knocking down MKP-5 enhanced the expression of these same apoptotic proteins (Figure 3E). Together, these results provided further evidence that MKP-5 protects Rin-m5f cells from PA-induced apoptosis and dysfunction.

### 2.4. MKP-5 Alleviates ERS and Autophagy Inhibition in PA-Stressed Rin-m5f Cells

**Figure 4:**
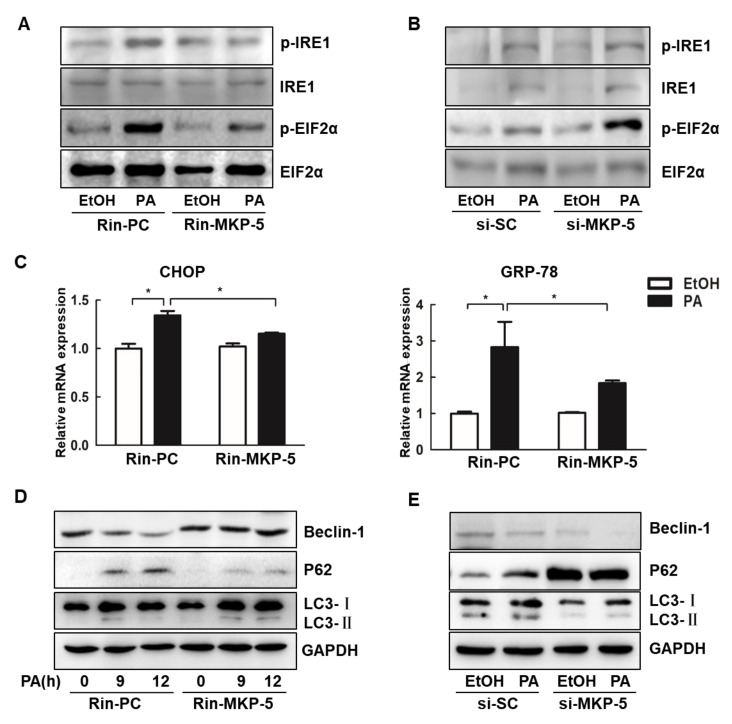
MKP-5 rescued PA-induced endoplasmic reticulum stress (ERS) and autophagy inhibition in Rin-m5f cells. (**A**) The levels of ERS-related proteins were analyzed by Western blotting in Rin-PC and Rin-MKP-5 cells treated with PA for 9 h. (**B**) The levels of ERS-related proteins in Rin-m5f cells transfected with si-SC or si-MKP-5 following PA treatment for 9 h were measured by Western blotting. (**C**) The mRNA levels of C/EBP-homologous protein (CHOP) and glucose-regulated protein-78 (GRP-78) in Rin-PC and Rin-MKP-5 cells following a 12 h PA treatment were detected by real-time PCR. (**D**) The levels of autophagy-related proteins were measured via Western blotting in Rin-m5f cells upon PA treatment for 9 and 12 h. (**E**) The levels of autophagy-related proteins were measured via Western blotting in Rin-m5f cells transfected with si-SC or si-MKP-5 at 9 h post-PA treatment. *, *p* < 0.05, EtOH vs. PA; Rin-PC vs. Rin-MKP-5.

Islet β-cells are often damaged by the ERS response in response to adverse conditions such as PA stimulation [32]. In the present study, we found that the overexpression of MKP-5 reduced the PA-induced expression of the ERS-related proteins phospho-eukaryotic initiation factor 2α (p-EIF2α) and phospho-inositol requiring enzyme 1 (p-IRE1) in Rin-m5f cells (Figure 4A). Furthermore, MKP-5 silencing enhanced PA-induced increases in the expression of p-IRE1 and p-EIF2α, potentially aggravating the ERS response in Rin-m5f cells (Figure 4B). In addition, the overexpression of MKP-5 significantly reduced the relative expression of ERS-related genes, including C/EBP-homologous protein (CHOP) and glucose- regulated protein-78 (GRP-78), both of which were induced by PA stimulation (Figure 4C). Islet β-cell dysfunction occurred not only as a result of direct PA-induced lipotoxicity but also due to the inhibition of autophagic flux that occurred upon PA treatment [9]. Beclin-1, a crucial regulator of autophagy, forms a complex with other autophagy proteins in order to mediate autophagosome assembly and to increase intracellular autophagic flux [33,34]. During autophagy, cytosolic microtubule-associated protein 1 light chain 3-I (LC3-I) is converted into microtubule-associated protein 1 light chain 3-II (LC3-II) and recruited to the autophagosomal membrane, with such conversion being an indicator of autophagosome formation [35,36]. In our study, the overexpression of MKP-5 rescued the PA-induced reduction in Beclin-1 observed at both 9 and 12 h post-treatment in Rin-m5f cells, whereas LC3-II levels were augmented only at 12 h post-PA treatment in Rin-MKP-5 relative to Rin-PC cells (Figure 4D). Levels of LC3-I did not vary as a function of MKP-5 overexpression or PA treatment. As an ubiquitin-binding protein, P62 has also been reported to be an autophagic substrate that is degraded by both the proteasome and within lysosomes [37,38]. MKP-5 overexpression inhibited the expression of P62 at both 9 and 12 h post-PA treatment (Figure 4D). Moreover, siRNA-mediated MKP-5 knockdown reduced the expression of Beclin-1 and LC3-II accumulation and increased the expression of P62 in Rin-m5f cells (Figure 4E). These results indicated that MKP-5 improves ERS and autophagy inhibition in PA-stressed Rin-m5f cells.

### 2.5. Overexpression of MKP-5 Enhances PA-Impaired Autophagic Flux in Rin-m5f Cells

**Figure 5:**
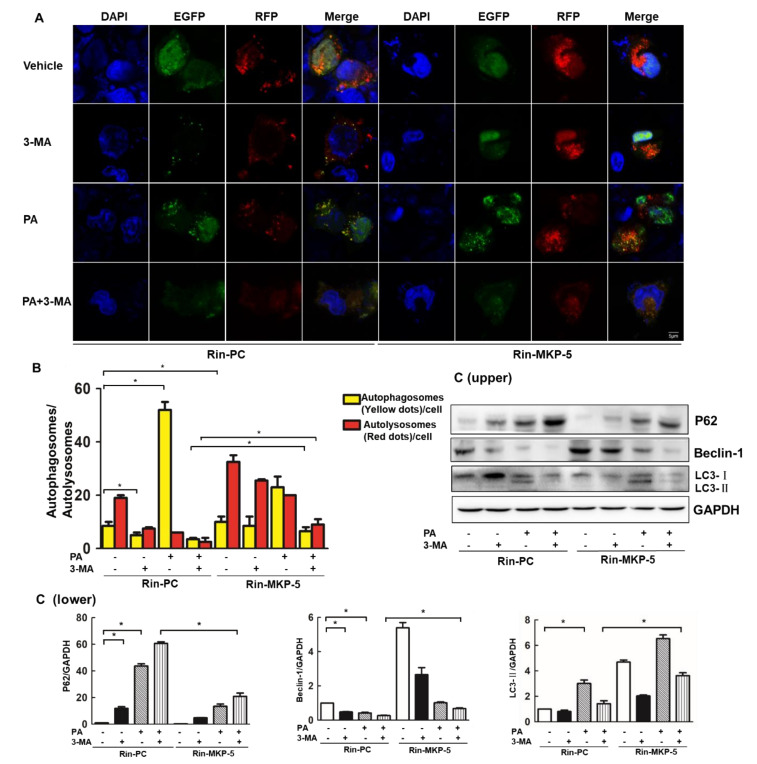
MKP-5 regulates the PA-induced impairment of autophagic flux in Rin-m5f cells. (**A**) Rin-PC and Rin-MKP-5 cells were transfected with the ptfLC3 plasmid for 48 h, after which they were treated with PA and 3-methyladenine (3-MA) for 9 h, and analyzed by laser scanning confocal fluorescence microscopy (scale bar = 5 µm). (**B**) Quantification of cells positive for red fluorescent protein (mRFP) (red) and mRFP/GFP (green fluorescent protein, yellow) in Rin-PC and Rin-MKP-5 cells transfected with ptfLC3 prior to a 9 h treatment with PA or 3-MA alone or in combination with one another. (**C**) The levels of autophagic proteins in Rin-m5f cells treated with PA ± 3-MA for 9 h were measured by Western blotting (upper) and quantitative analysis was conducted using Image J (lower). *, *p* < 0.05, 3-MA vs. Vehicle; PA vs. Vehicle; Rin-PC vs. Rin-MKP-5.

Autophagy maintains cellular metabolism and acts as a protective factor in cellular survival by eliminating unnecessary cellular organelles [39]. 3-Methyladenine (3-MA) is a phosphoinositide 3-kinase (PI3K) inhibitor that is known to inhibit the formation of autophagosomes and widely used as an autophagy inhibitor [40]. To further investigate how MKP-5 regulates autophagic flux in Rin-m5f cells upon PA exposure, we used the ptfLC3 tandem fluorescent reporter plasmid to transfect Rin-PC or Rin-MKP-5 cells following PA and 3-MA treatment. The ptfLC3 plasmid initially encodes both green fluorescent protein (GFP) and red fluorescent protein (mRFP), resulting in an overlapping yellow fluorescent signal in autophagosomes. The GFP protein is degraded in response to lysosomal fusion and associated increases in acidic conditions, whereas the RFP signal is maintained. Therefore, in merged fluorescent images, autophagosomes appear yellow, whereas autolysosomes appear red. The enhancement of autophagic flux results in an increase in the number of autolysosomes, whereas the suppression of autophagic flux can result in reduced autolysosome numbers and increased autophagosome numbers [41]. As shown in Figure 5A, PA treatment in Rin-PC cells resulted in an increase in the numbers of autophagosomes within cells as well as a decrease in autolysosome levels, suggesting that PA disrupted autophagic flux prior to the conversion of autophagosomes to autolysosomes. Moreover, 3-MA treatment of Rin-PC cells resulted in the visible loss of red and yellow LC3 puncta compared with vehicle control, especially when used in combination with PA treatment, illustrating that concomitant PA and 3-MA treatment resulted in a near complete disruption of autophagic flux (Figure 5A,B). Consistent with our hypothesis, the numbers of red puncta increased significantly in the Rin-MKP-5 vehicle control cells relative to Rin-PC cells, whereas no changes in the frequencies of yellow puncta were observed (Figure 5A,B), indicating that MKP-5 may enhance basal autophagic flux in Rin-m5f cells. Furthermore, red puncta displayed an increase in the presence of PA-treated Rin-MKP-5 cells, especially in the presence of 3-MA compared with Rin-PC cells, which accounts for MKP-5 overexpression that may improve both the formation and the conversion of autophagosomes inhibited by 3-MA or PA (Figure 5A,B). We further explored the molecular mechanisms underlying these results. P62 expression was enhanced in Rin-PC cells treated with 3-MA or PA, and the enhancement was more profound when cells were treated with both of these compounds, whereas the overexpression of MKP-5 reduced the expression of P62 in each treatment relative to that observed in Rin-PC cells. In addition, the overexpression of MKP-5 rescued PA- and 3-MA-induced impairments in Beclin-1 levels. In PA-treated Rin-PC cells, increased LC3-II expression was relieved by 3-MA treatment. Importantly, MKP-5 overexpression enhanced the PA-induced increases in LC-3-II expression in the presence or absence of 3-MA (Figure 5C). Collectively, we concluded that the overexpression of MKP-5 relieved the PA-induced impairment of autophagic flux observed in Rin-m5f cells. 

### 2.6. MKP-5 Protects Primary Islet Cells against HFD-Induced ERS, Apoptosis, Dysfunction, and Autophagy Inhibition

**Figure 6:**
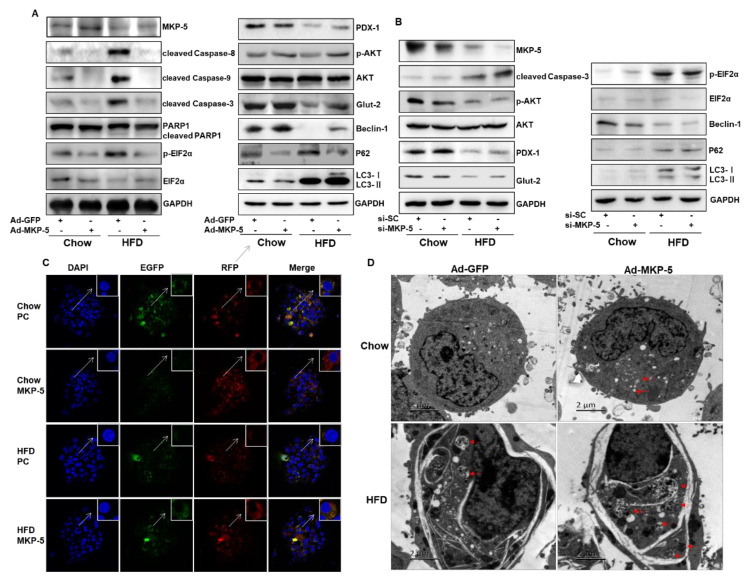
MKP-5 mediates obesity-induced apoptosis, ERS, dysfunction, and autophagy inhibition in primary mouse islet cells. A, B. Mouse primary islet cells were isolated from HFD or chow diet-fed mice and then infected with Ad-MKP-5/Ad-GFP (**A**) or transfected with si-SC/si-MKP-5 (**B**) (male mice, *n* = 7 per group). Then, the expression of MKP-5, apoptotic proteins, ERS-related proteins, islet cell functional proteins, and autophagy-related proteins were analyzed by Western blotting. (**C**) Autophagy was monitored in primary islet cells transfected with ptfLC3 and pcDNA3.1/pcDNA3.1-MKP-5 for 72 h. LC3-labeled representative fluorescent images were observed by confocal fluorescence microscopy. (**D**) Autolysosomes indicated by red arrows in primary islet cells from HFD or chow diet-fed mice were observed by TEM (scale bar = 2 µm).

To further assess the importance of MKP-5 in primary islet cells, we isolated islet cells from mice fed with either a HFD or a chow diet. Then, these islet cells were infected with recombinant adenoviruses overexpressing MKP-5 (Ad-MKP-5) or GFP (Ad-GFP). As shown in Figure 6A, the expression level of MKP-5 was decreased in primary islet cells from HFD-fed mice as compared to that in chow-fed mice. Moreover, HFD increased the expression of mitochondrial apoptotic proteins, including cleaved Caspase-9, cleaved Caspase-3, and cleaved PARP-1 in primary islet cells infected with Ad-GFP, which is consistent with the PA treatment results shown in Figure 1 (Figure 6A). Levels of cleaved Caspase-8, a vital apoptotic protein related to the death receptor pathway, were also increased by HFD, which was distinct from our assessment of PA-treated Rin-m5f cells, wherein Caspase-8 activation was not affected by lipotoxicity (Figure 6A). Importantly, adenovirus-mediated overexpression of MKP-5 abolished these HFD-mediated increases in apoptotic protein levels in primary islet cells (Figure 6A). In addition, Ad-MKP-5 alleviated the HFD-mediated upregulation of p-EIF2α in these primary cells relative to Ad-GFP (Figure 6A). Concomitantly, Ad-MKP-5 significantly increased the levels of p-AKT, Glut-2, and PDX-1 that had been inhibited by HFD (Figure 6A). Moreover, HFD upregulated the levels of the autophagy-related proteins P62 and LC3-II, and it downregulated the levels of Beclin-1 in primary islet cells infected with Ad-GFP (Figure 6A). Notably, Ad-MKP-5 was able to promote the expression of Beclin-1 and inhibit the expression of P62 in both HFD and chow groups as compared with Ad-GFP treatment. Nevertheless, Ad-MKP-5 seemed to induce a slight increase in LC3-II levels in primary islet cells from HFD-fed mice relative to Ad-GFP, potentially due to HFD-induced enhancement of LC3-II signaling. 

To further determine the protective role of MKP-5 in mouse islets, we knocked down the expression of MKP-5 via siRNA in primary islet cells separated from HFD or chow diet-fed mice. Consistent with previous studies, HFD increased the expression of the apoptotic protein cleaved Caspase-3, and this was further aggravated by MKP-5 knocked down. In addition, the expression of p-AKT was inhibited by MKP-5 knockdown in islet cells from both chow and HFD-fed mice. Unexpectedly, the two other islet functional proteins PDX-1 and Glut-2 were not affected by MKP-5 knockdown in islet cells from either chow or HFD-fed mice, and further study will be required to understand the significance of this result. HFD upregulated the levels of the ERS-related protein p-EIF2α in islet cells treated with si-SC relative to the chow group (Figure 6B), which is consistent with the observed results in Rin-m5f cells treated with PA. Furthermore, the inhibition of MKP-5 was associated with p-EIF2α activation in the chow group, while the loss of MKP-5 did not aggravate p-EIF2α expression in the HFD group, potentially due to its robust HFD-associated induction (Figure 6B). Concomitantly, siRNA-induced MKP-5 knockdown was able to decrease the levels of the autophagy-related protein Beclin-1 in both chow and HFD groups treated with si-SC, although it had no effect on the levels of P62 or LC3-II (Figure 6B). Collectively, these results further suggested that MKP-5 plays a crucial role in the regulation of ERS, apoptosis, cell dysfunction, and autophagy in primary islets in response to PA. Next, we examined the effect of MKP-5 on autophagy in primary islet cells. More diffuse red staining was observed in islet cells in which MKP-5 was overexpressed compared to PC controls in the chow-diet group. Moreover, MKP-5 overexpression partially rescued the observed reductions in yellow puncta observed in HFD-treated islet cells relative to chow controls, suggesting that MKP-5 alleviated HFD-induced autophagic suppression (Figure 6C). In contrast, enlarged and more numerous autolysosomes were evident in primary islet cells from HFD-treated animals relative to those in the chow group (Figure 6D). MKP-5 overexpression increased the number of cytoplasmic autolysosomes, as visualized via TEM, with this being most evident in HFD-induced mouse islet cells (Figure 6D). This further suggested that MKP-5 overexpression was able to enhance the autophagic flux in these cells. These results provide more evidence supporting a role for MKP-5 as a protective factor preventing islet cell apoptosis, ERS, dysfunction, and autophagy inhibition in the context of obesity.

### 2.7. MKP-5 Regulates the Apoptosis and Dysfunction of Islet Cells through the JNK and P38 Pathways

**Figure 7:**
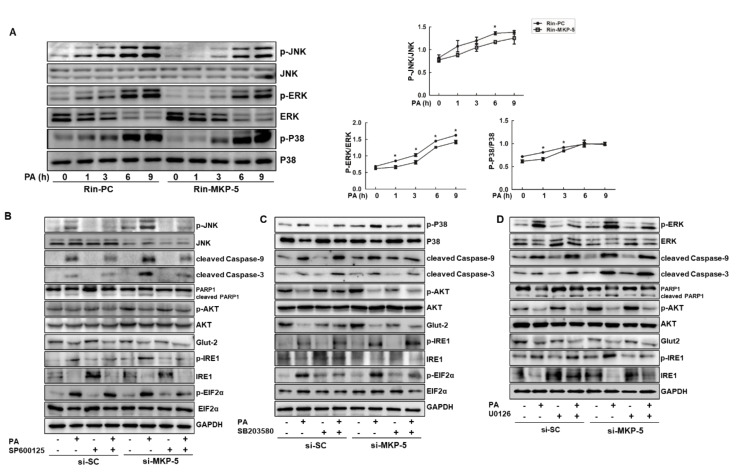
c-Jun amino-terminal kinase (JNK) and P38 are implicated in MKP-5-mediated regulation of Rin-m5f cell apoptosis and dysfunction in response to PA. (**A**). Rin-PC and Rin-MKP-5 cells were stimulated with PA for the indicated period of time, and then the levels of phosphorylated and total JNK, P38, and extracellular-regulated kinase (ERK) were detected by Western blotting. (**B**). Protein band intensity was quantified using the Image J software, and phosphorylated protein bands were normalized to their respective total protein levels. Rin-PC and Rin-MKP-5 cells were pretreated with SP600125 (50 μM) (**C**), SB230580 (5 μM) (**D**), or U0126 (5 μM) for 1 h and then stimulated with corresponding inhibitor and PA for 9 h. The levels of MAPKs, apoptotic proteins, ERS-related proteins, and functional proteins were measured by Western blotting, and GAPDH was used as a loading control. All data are means ± SEM. *, *p* < 0.05, Rin-PC vs. Rin-MKP-5.

We next assessed whether MKP-5 affected the activation of MAPKs, including JNK, ERK, and P38 MAPK, in Rin-m5f cells. As shown in Figure 7A, the phosphorylation levels of JNK, ERK, and P38 gradually increased in Rin-PC cells after PA treatment, whereas MKP-5 overexpression inhibited the PA-induced activation of these MAPKs (Figure 7A). The levels of p-JNK/JNK in Rin-MKP-5 cells were significantly lower at 6 h relative to in Rin-PC cells upon PA stimulation. MKP-5 overexpression also markedly reduced the phosphorylation of ERK, which was activated by PA at 1, 3, 6, and 9 h. The upregulation of p-P38/P38 induced by PA in Rin-m5f cells was also clearly inhibited by MKP-5 overexpression at 1 and 3 h post-stimulation (Figure 7B) relative to Rin-PC cells. To further determine the specific MAPK pathways involved in MKP-5-mediated regulation of islet cell apoptosis and dysfunction in the context of obesity, we pretreated Rin-m5f cells with SP600125 (JNK inhibitor), SB203580 (P38 inhibitor), or U0126 (ERK inhibitor) for 1 h, which was followed by transfection with si-MKP-5 or si-SC and PA exposure. Upon MKP-5 knockdown, the phosphorylation of JNK, P38, and ERK was increased both basally and following 9 h of PA treatment, suggesting that the P38, ERK, and JNK MAPKs may be targets of MKP-5 in Rin-m5f cells (Figure 7C). SP600125 reduced the PA-mediated upregulation of apoptotic proteins, including cleaved Caspase-3 and cleaved Caspase-9 in MKP-5 knockdown Rin-m5f cells (Figure 7C). However, SP600125 did not affect the expression of the functional p-AKT and Glut-2 proteins, which had been downregulated in response to PA treatment. These results suggested that the JNK mediates MKP-5-mediated regulation of apoptosis but not dysfunction in PA-treated Rin-m5f cells. The P38 inhibitor SB203580 did not alter the PA-mediated induction of apoptotic proteins in Rin-m5f cells, whereas this inhibitor did reverse the PA-mediated changes in p-AKT and Glut-2 expression in Rin-m5f cells (Figure 7D). This indicated that P38 is involved in the MKP-5-mediated regulation of PA-induced Rin-m5f cell dysfunction rather than apoptosis. While we did find that MKP-5 overexpression could reduce PA-mediated ERK activation, U0126 did not alter the effects of PA on the expression of apoptotic or functional proteins in Rin-m5f cells regardless of si-MKP-5 transfection status (Figure 7E). This suggests that the ERK pathway is not involved in the MKP-5-mediated regulation of the apoptosis and dysfunction of islet cells. Unexpectedly, knocking down MKP-5 increased levels of p-AKT in the vehicle control group, which may be the result of a feedback regulation cycle and warrants further study. Collectively, these results demonstrate that MKP-5 mediates the apoptosis and dysfunction of islet cells in response to PA through the JNK and P38 pathways. 

### 2.8. MKP-5 Protects Rin-m5f Cells from Lipotoxicity via the Autophagy Pathway 

**Figure 8:**
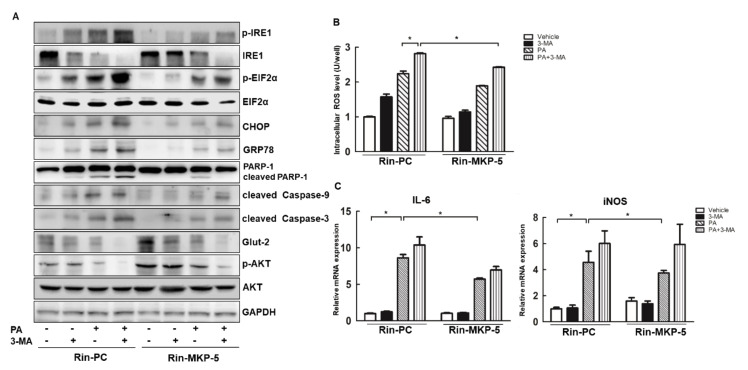
MKP-5 regulates PA-induced autophagy in Rin-m5f cells. (**A**) The levels of ERS-, apoptosis-, dysfunction-, and functional-related proteins in Rin-PC and Rin-MKP-5 cells treated with PA and 3-MA for 12 h were measured by Western blotting. (**B**) Levels of 3-MA and PA-induced intracellular ROS in Rin-PC and Rin-MKP-5 cells were determined using a fluorescence microplate reader by measuring DCFH-DA-fluorescence. (**C**) Expression of the inflammation-related genes iNOS, IL-6, and MCP-1 were detected by real-time PCR in Rin-m5f cells following PA and 3-MA exposure for 9 h. *, *p* < 0.05, EtOH vs. PA; Rin-PC vs. Rin-MKP-5.

Recently, MKP has been suggested to regulate autophagy [26]. To further explore whether MKP-5 would regulate apoptosis and dysfunction by autophagy pathway, Rin-PC and Rin-MKP-5 cells were treated with both PA and with the autophagic inhibitor 3-MA for 9h. As shown in Figure 8A, levels of the apoptotic proteins cleaved PARP-1, cleaved Caspase-9, and cleaved Caspase-3 were increased upon 3-MA-meditated autophagic inhibition in the absence of PA in Rin-PC and Rin-MKP-5 cells. PA-induced increases in the levels of these proteins were markedly increased after exposure to 3-MA in Rin-PC cells, while MKP-5 overexpression alleviated the 3-MA-induced exacerbation of mitochondrial apoptosis. In addition, PA induced the activation of ERS-related proteins including p-IRE1, p-EIF2α, CHOP, and GRP-78, and this was aggravated by 3-MA treatment (Figure 8A). Moreover, the upregulation of MKP-5 owned an effect on eliminating the above ERS-related proteins whether PA existed or not, especially in the presence of 3-MA against Rin-PC cells. Therefore, we concluded that MKP-5 improved the functionality of the autophagic pathway in order to drive mitochondrial apoptosis and ERS in response to PA in Rin-m5f cells.

In addition, we also observed that 3-MA treatment inhibited the expression of p-AKT and Glut-2, especially in the presence of PA in Rin-PC cells. Consistent with this, MKP-5 overexpression reversed 3-MA-induced autophagic inhibition as evidenced by changes in Glut-2 and p-AKT levels in the presence or absence of PA treatment (Figure 8A). This suggests that MKP-5 alleviated PA-induced apoptosis and dysfunction via rescuing autophagic signaling. In accordance with this increased point on obesity-related diabetes, the role of autophagy in the regulation of inflammatory and oxidative stress started to emerge [13,42,43]. We found that 3-MA treatment promoted ROS production in the presence or absence of PA in Rin-PC cells, while MKP-5 overexpression rescued 3-MA-mediatedincreases in ROS levels in PA-treated cells (Figure 8B). We have also shown that MKP-5 alleviated PA-induced inflammation in Rin-m5f cells (Figure 2D). Thus, our results confirm that autophagy was involved in this process. Surprisingly, 3-MA treatment alone did not alter the levels of inflammatory cytokines, such as IL-6 and iNOS in Rin-PC cells (Figure 8C). Nevertheless, MKP-5 overexpression reduced the levels of IL-6 and iNOS induced by the combination 3-MA + PA treatment in Rin-MKP-5 cells (Figure 8C). Collectively, these above results suggest that MKP-5 mediates PA-induced apoptosis, ERS, dysfunction, oxidative stress, and inflammation via the autophagy pathway.

## 3. Discussion

Obesity is a major risk factor for diabetes, and it is associated with a state of chronic inflammation and elevated levels of FFAs. As a component of these FFAs, PA has been reported to be involved in the development of apoptosis and the dysfunction of islet β-cells. Increasing oxidative stress and ERS are also known to be involved in this process [44,45], particularly in the MAPK-dependent apoptosis and dysfunction of islet cells [46]. As a member of the MKP family, MKP-5 is closely associated with insulin resistance and inflammation [28]. In our study, we explored the role of MKP-5 in the regulation of the apoptosis and dysfunction of islet β-cells related in the context of obesity. We found that MKP-5 was downregulated in mice fed a HFD, and then, we modulated MKP-5 expression in islet cells via overexpression and siRNA-mediated knockdown approaches. The elevated expression of MKP-5 leads to a decline in the levels of apoptotic proteins, including Caspase-9, Caspase-3, and PARP-1, which are involved in the mitochondrial pathway-activated in response to PA in Rin-m5f cells. MKP-5 also reduced the degree of PA-induced ERS in Rin-m5f cells by reducing the PA-induced increases in the expression of p-IRE1 and p-EIF2α, indicating that MKP-5 may be implicated in the IRE1 and PERK pathway during ERS responses. In addition, the overexpression of MKP-5 upregulated the relative expression of Bcl-2 and downregulated the relative expression of CHOP upon PA treatment, thus indicating that the PERK/elF2α/ATF4 pathway may be an upstream regulator of CHOP, further impacting Bcl-2 and driving mitochondrial loss and eventual apoptosis. Furthermore, knockdown experiments provided further evidence for the protective role of MKP-5 in islet cells in response to lipotoxicity. SP600125, a JNK inhibitor, markedly alleviated the observed increases in levels of cleaved PARP-1, cleaved caspase-9, and cleaved caspase-3 in MKP-5-knockdown Rin-m5f cells, while also decreasing the degree of PA-induced IRE1 phosphorylation. IRE1 is reported to be an upstream regulator of JNK during ERS-associated apoptotic cell death [47], and our results therefore suggest that the IRE1/JNK pathway may be involved in the regulation of the protective activity of MKP-5 against apoptosis in PA-treated islet cells. In summary, MKP-5 may relieve lipotoxicity-induced islet cell apoptosis via the JNK pathway.

As T2DM progresses, so too does the dysfunction and apoptosis of islet β-cells. The failure of GSIS in these cells is associated with the impairment of Glut-2 and p-AKT expression [48]. In addition, PDX-1 is a vital transcription factor known to be required for the maintenance of GSIS and the function of islet cells [49]. Likewise, GSIS is also impacted by GLP-1R, a receptor expressed on β-cells, which controls blood sugar levels by enhancing insulin secretion, further protecting against lipotoxic apoptosis in islet β-cells [50]. In the present study, we found that the overexpression of MKP-5 both relieved the PA-mediated inhibition of GSIS as well as rescued the expression of Glut-2, p-AKT, PDX-1, and GLP-1R upon PA stimulation. In contrast, the silencing of MKP-5 further exacerbated the inhibition of GSIS and further reduced already low levels of AKT phosphorylation and Glut-2 in response to PA stimulation. Notably, the treatment of cells with a P38 inhibitor was sufficient to restore GSIS and p-AKT/Glut-2 expression in cells treated with PA in which MKP-5 had been knocked down. Taken together, these results suggest that MKP-5 can alleviate the obesity-induced dysfunction of islet cells via the P38 pathway. 

In this ex vivo study, we found that HFD drove apoptosis and dysfunction in mouse primary islet cells. In particular, we found that HFD played a much broader role in inducing the apoptosis of mouse primary islet cells as compared to the role of PA in inducing the apoptosis of Rin-m5f cells. HFD was able to trigger a total of three apoptotic pathways in these primary cells, including the mitochondrial pathway, the ERS pathway, and the death receptor pathway. Importantly, adenovirus-mediated MKP-5 overexpression in primary mouse islet cells diminished HFD-induced apoptosis via these three pathways. In addition, elevated MKP-5 expression also rescued lipotoxic dysfunction in these primary islet cells. Thus, these data provide further evidence that MKP-5 protects islet cells from lipotoxicity-induced apoptosis and dysfunction. 

We previously found that PA treatment was able to increase the inflammatory profile and trigger the ERS response in adipocytes [13]. The resultant chronic inflammatory response is associated with extensive free radical production by immune cells, thereby causing substantial oxidative stress [51]. ROS can in turn activate the transcription factor nuclear factor kappa B (NF-kB) and promote the expression of TNF-α and IL-6, driving a cellular inflammatory response [52]. A recent study found that MKP-5 contributed to reducing inflammatory cytokines IL-6 and TNF-α and generating ROS in endothelial cells [53]. Herein, we confirmed that the overexpression of MKP-5 repressed the intracellular ROS generation and the levels of inflammatory-related genes, including IL-6, TNF-α, and MCP-1, induced by PA in Rin-m5f cells. In addition, the overexpression of MKP-5 inhibited the PA-induced production of intracellular ROS and its associated genes, P22phox and Nox-4. One recent study found that ROS are able to inhibit PI3K/AKT insulin signaling and mitochondrial function, inducing the apoptosis of islet β-cells [54]. Similarly, MKP-5 may impair the release of inflammatory factors and the production of ROS, thereby reducing the apoptosis and dysfunction of islet cells under lipotoxic conditions.

Autophagy has been found to play a critical role in cellular metabolism and can also maintain the function of mitochondria and the endoplasmic reticulum. Therefore, impaired autophagy caused by aging and obesity with lipid overload can lead to β-cell dysfunction [55]. Impaired autophagic flux was also found to be closely associated with lipotoxicity-induced β-cell dysfunction, endoplasmic reticulum stress, oxidative stress, inflammation, and mitochondrial dysfunction [10]. Herein, we demonstrated that the 3-MA- or PA-induced autophagic inhibition was alleviated by MKP-5 overexpression. Most importantly, MKP-5 overexpression was able to suppress apoptosis, dysfunction, endoplasmic reticulum stress, oxidative stress, and inflammation in Rin-m5f cells. Furthermore, we confirmed that the overexpression of MKP-5 alleviated impaired autophagic flux by reducing the inhibition of autophagosome-to-autolysosome conversion in Rin-m5f cells. This result was further confirmed based on changes in the levels of the autophagic markers P62, Beclin-1, and LC3-II in these cells. MAPK signaling has been reported to be involved in the regulation of autophagy [20]. Notably, the treatment of Rin-m5f cells with a JNK or P38 inhibitor was sufficient to normalize the expression of autophagic proteins that had been altered by 3-MA and PA, especially in the context of MKP-5 overexpression. However, it still remains uncertain as to whether or not HFD plays a role in inhibiting autophagic flux [56,57]. In our ex vivo study, MKP-5 also rescued HFD-inhibited autophagic flux by increasing both the formation of autophagosomes and the degradation of autolysosomes. Therefore, the results suggest that MKP-5 can alleviate obesity-inhibited autophagy in islet cells via the JNK and P38 pathway. 

In summary, in this study, we have demonstrated that MKP-5 reduced the lipotoxic apoptosis and the dysfunction of these cells via the autophagy pathway. In addition, the JNK and P38 MAPK pathways were also involved in mediating autophagic flux, apoptosis, and dysfunction in islet cells. While MKP-5 also reduced the phosphorylation of ERK in Rin-m5f cells, no apoptosis, functional proteins, or autophagy-related proteins were impacted by treatment with an ERK inhibitor, suggesting that this pathway regulation does not have functional consequences in this context. In conclusion, MKP-5 negatively regulates lipotoxicity-induced apoptosis and dysfunction by inhibiting JNK and P38 activation in islet cells. This finding offers new insights that may be relevant to the field of T2DM drug development.

## 4. Materials and Methods

The Rin-m5f and 293A cell lines were obtained from the American Type Tissue Culture Collection (Manassas, VA, USA). RPMI-1640 medium was from Hyclone (Logan, UT, USA). Fetal bovine serum (FBS) was purchased from Gibco (Waltham, MA, USA). Opti-minimal essential medium (MEM) reduced serum and Lipofectamine 2000 were purchased from Invitrogen Life Technology (Carlsbad, CA, USA). D-(+)-Glucose, palmitic acid (PA), and fatty acid-free bovine serum albumin (BSA) were supplied by Sigma (Louis, MO, USA). The CellTiter96^®^ AQueous One Solution Cell Proliferation Assay kit was from Promega (Madison, WI, USA). The Annexin-V-FLUOS staining kit, complete protease inhibitor cocktail tablets, and FastStart Universal SYBR Green Master with ROX were purchased from Roche Diagnostics (Indianapolis, IN, USA). The Hochest 33,258 and Caspase-3 activity assay kits were from Beyotime Institute of Biotechnology (Shanghai, China). JNK inhibitor (SP600125), ERK inhibitor (U0126), and autophagy inhibitor (3-MA) were purchased from MedChemExpress (Monmouth Junction, NJ, USA). P38 inhibitor (SB203580) was from Calbiochem (Darmstadt, Germany). Antibodies against phospho-JNK, JNK, phospho-ERK1/2, ERK1/2, phospho-P38, P38, cleaved Caspase-3, AKT, EIF2α, DUSP10/MKP-5, CHOP, GRP-78, PDX-1, Beclin-1, P62, and LC3 were purchased from Cell Signaling Technology (Beverly, MA, USA). Antibodies against cleaved Caspase-9, cleaved Caspase-8, PARP-alpha, phospho-AKT, Glut-2, phospho-EIF2α, phospho-IRE1, IRE1 and the ECL kit (femtogram) were from Affinity Biosciences (Cincinnati, OH, USA). Antibodies against GAPDH and horseradish peroxidase (HRP)-conjugated secondary antibodies against rabbit, mouse, and goat primary antibodies were purchased from Santa Cruz Biotechnology (Danvers, MA, USA). Enhanced chemiluminesence reagent was purchased from Pierce Biothenology (Rockford, IL, USA). A PrimeScript RT reagent kit with gDNA Eraser and Premix Ex Taq^TM^ PCR kit were obtained from TaKaRa (Dalian, China). The Pierce BCA protein assay kit was purchased from Thermo Scientific (Waltham, MA, USA). The plasmid of ptfLC3 was provided by Addgene (Cambridge, MA, USA)

### 4.1. Cell Culture and Stimulation

Rin-m5f cells were maintained in RPMI-1640 containing 10% FBS at 37 °C in a humidified 5% CO_2_ incubator. The 293A cells were cultured in dulbecco’s modified eagle medium (DMEM) supplemented with 10% FBS. For PA exposure, PA was dissolved in ethanol at 50 mM as a stock solution and then used at a final concentration of 0.5 mM. Unless otherwise specified, Rin-m5f cells were serum starved overnight and then cultured in media containing 0.5 mM PA or the same volume of ethanol conjugated to 0.5% fatty acid-free BSA. 

### 4.2. Generation of Stable MKP-5-Expressing Rin-m5f Cells 

Rin-m5f cells were transfected with the pcDNA3.1-MKP-5 or pcDNA3.1 plasmids using Lipofectamine 2000 and then underwent antibiotic selection in media containing 400 μg/mL G418 for 3 weeks. MKP-5 overexpressing Rin-m5f cells were thereafter maintained in RPMI-1640 medium containing 10% FBS and 200μg/mL G418. Rin-m5f cells stably transfected with pcDNA3.1 were termed Rin-PC cells, while pcDNA3.1-MKP-5 stably transfected cells were termed Rin-MKP-5 cells.

### 4.3. Construction of a Recombinant Adenovirus

The recombinant adenoviral shuttle plasmid pHBAd-MCMV-MKP-5 and the skeleton plasmid pHBAd-BHGloxΔE1, 3 Cre (Hanbio Biotechnology, Shanghai, China) were co-transfected into 293A cells to facilitate adenovirus production and tittering. Mouse primary islets were isolated from C57/BL-6J mice fed a chow diet or a HFD, plated in 6-well plates, and infected with 100 μL (1 × 10^9^ plaque-forming units/mL) of the adenovirus encoding MKP-5 (Ad-MKP-5) for 6 h, after which these cells were cultured in RPMI-1640 containing 20% FBS for an additional 48 h. An adenovirus expressing GFP (Ad-GFP) was used as a negative control.

### 4.4. Small Interfering RNA (siRNA) Transfection 

Rin-m5f cells were transfected with small interfering RNA (siRNA) against MKP-5 (si-MKP-5, Stealth RNAi siRNA, Catalog #1330001) or a negative control scramble siRNA (si-SC, Stealth RNAi siRNA Negative Control, Catalog #12935300) using Lipofectamine RNAiMAX according to the manufacturer’s instructions in order to achieve a final siRNA concentration of 20 nM. For the experiments involving MAPK inhibition, Rin-m5f cells transfected with these siRNAs were preincubated with SP600125 (JNK inhibitor, 50 μM), SB203580 (P38 inhibitor, 5 μM), or U0126 (ERK inhibitor, 5 μM) for 1 h; then, they were stimulated with PA plus the above inhibitors for another 9 h, and proteins were prepared for Western blotting. 

### 4.5. Cell Proliferation Assay

Cell viability was evaluated via the 3-(4,5-dimethylthiazol-2-yl)-5-(3-carboxymethoxyphenyl)-2-(4-sulfophenyl)-2H-tetrazolium, inner salt (MTS) method using a CellTiter96^®^ Aqueous assay proliferation kit. Briefly, Rin-m5f cells were plated at 5 × 10^3^ cells/well in 96-well plates and incubated overnight, and then, they were treated with PA for 12 or 24 h. Following this treatment, 20 μL of MTS solution was added to each well, and the plate was incubated for another 1 h at 37 °C in a humidified 5% CO_2_ atmosphere. Then, the absorbance of each well was measured at 490 nm with a microplate reader.

### 4.6. Annexin-V-FLUOS/Propidium Iodide (PI) Analysis

The Annexin-V-FLUOS kit was used for the quantitative analysis of apoptotic cells. Briefly, Rin-m5f cells were collected after PA treatment for 24 h, and were washed with phosphate buffer saline (PBS) twice, resuspended in an Annexin-V-FLOUS and PI labeling solution, and incubated for 15 min at 25 °C. Then, the ratio of apoptotic cells was determined by flow cytometry, and data were analyzed using the CellQuest software (BD, Franklin Laked, NJ, USA).

### 4.7. Caspase-3 Activity Analysis

Caspase-3 activity was measured with a caspase-3 assay kit according to the manufacturer’s protocol. Rin-m5f cells were lysed following a 24 h PA stimulation and were centrifuged at 12,000 rpm for 15 min at 4 °C. Then, cell lysates were incubated in reaction buffer containing the caspase-3 fluorogenic substrate Ac-DEVD-pNA at 37 °C for 2 h. Then, the absorbance of p-nitroanilide (pNA) cleaved by caspase-3 was measured at 405 nm with a microplate reader (Bio-Tek, Winooski, VT, USA).

### 4.8. Measurement of Glucose Stimulated Insulin Secretion (GSIS)

Rin-m5f cells were stimulated with PA for 9 h. To measure insulin release, cells were first pre-incubated in HEPES-balanced Krebs-Ringer bicarbonate buffer (KRBH) with 2.8 mM glucose for 1 h, and then, they were incubated with KRBH in the presence of 2.8 mM or 16.7 mM glucose for 2 h at 37 ℃, 5% CO_2_. Then, supernatants were harvested and assayed using the Rat IN ELISA kit (Elabscience, Wuhan, China) in accordance with the manufacturer’s instructions. Then, Rin-m5f cells were lysed to facilitate protein quantification. Levels of secreted insulin from Rin-m5f were normalized to total protein content.

### 4.9. Measurement of Intracellular Reactive Oxygen Species (ROS)

The level of intracellular ROS was quantified by using ROS assay kit according to the manufacturer’s instructions (Beyotime, China). Briefly, after PA treatment for 24 h, Rin-m5f cells were washed with PBS thrice and kept in dark for 20 min at 37 °C following incubation with 10 μM of DCFH-DA. The qualitative analysis of ROS generation was measured using a fluorescence microscope with excitation at 488 nm and emission at 525 nm.

### 4.10. RNA Preparation and Quantitative Real-Time PCR

Total RNA was extracted using the Trizol reagent and was reverse transcribed using oligo primers according to the manufacturer’s recommended protocol. Then, quantitative real-time PCR reactions were performed using SYBR Green Master with ROX on a StepOnePlus system (Applied Biosystems, Carlsbad, CA, USA). Primers for the detection of mRNA expression were synthesized by Sangon Biotech (Shanghai, China), and the sequences are listed in Table 1. The relative mRNA levels normalized to GAPDH were calculated according to the comparative 2^−ΔΔCt^ method.

### 4.11. Western Blotting

Cells or tissue samples were lysed with ice-cold lysis buffer supplemented with complete protease inhibitor cocktail, and protein concentrations were measured using a BCA protein assay kit. Then, the cell lysates were separated on 8–15% SDS-PAGE gels and transferred onto polyvinylidene fluoride (PVDF) membranes that were subsequently blocked with 5% BSA. Then, the membranes were incubated with appropriate primary antibodies overnight, followed by appropriate secondary antibodies. Then, the protein bands were imaged using enhanced chemiluminescence (ECL) reagents and quantified with the Image J software.

### 4.12. Mouse Models and Mouse Primary Islets Isolation

Five-week-old male C57BL6/J mice (HUAFUKANG Bioscience, Beijing, China) were housed 8 per cage under a light/dark cycle of 12 h with free access to water and were fed either a normal chow diet or a high-fat diet (HFD, 60% kcal from fat, HUAFUKANG Bioscience, Beijing, China) continuously for 16 weeks. All animal experiments were performed in accordance with the guidelines for the Care and Use of Experimental Animals of Jilin University and were approved by the Animal Experiment Ethics Committee of Jilin University (number of permit: 20190048). At the end of study, mice were sacrificed by CO_2_ asphyxiation. As reported previously, primary islet cells were isolated from 21-week-old C57BL/6J male mice via collagenase digestion [30]. Briefly, pancreas samples were weighed, cut into pieces in D-Hanks, and digested with 1 mg/mL type V collagenase at 37 °C in a shaker at 80 rpm for 30 min. Then, tissue suspensions were filtered through 550 μm mesh filters, centrifuged on Ficoll 400 (Alfa Aesar, MA, USA) gradient (27.0%, 23.0%, 20.5%,11.0%) to separate islets from acini, and then cultured in DMEM containing 20% FBS at 37 °C under 5% CO_2_ conditions. Further, islet cells from HFD or chow-fed mice were infected with the recombinant adenoviruses Ad-MKP-5/Ad-GFP (1 × 10^9^ plaque-forming units/mL) or si-MKP-5/si-SC for 48 h before protein extraction. 

### 4.13. Laser Scanning Confocal Microscopy Analysis

The Rin-PC and Rin-MKP-5 cells seeded in the coverslips were transfected with the plasmid of ptfLC3 for 48 h alongside with the treatment of 3-MA (5 mM) in the presence or absence of PA for 9 h. The islets isolated from HFD or chow-fed mice were also seeded in the coverslips. After a 72-h incubation, the coverslips were fixed with 4% paraformaldehyde for 30 min. After wiping off the additive paraformaldehyde with PBS, the coverslips were observed under Laser Scanning Confocal Microscope (LSM 780, Carl Zeiss, Germany) for GFP-RFP-LC3 puncta formation.

### 4.14. Transmission Electron Microscopic Observation

Primary mouse islets from HFD or chow-fed mice were plated in 6-well plates and infected with the recombinant adenoviruses Ad-MKP-5/Ad-GFP (1 × 10^9^ plaque-forming units/mL) for 6 h, after which these islets were cultured in RPMI-1640 containing 20% FBS for an additional 48 h. The islets were harvest in 0.1 M Sorensen phosphate buffer (pH 7.2). The specimens were fixed in 2.5% glutaraldehyde for 24 h and then washed in Sorensen’s phosphate buffer again. Hereafter, the specimens were sent to another fixation by 2% osmium tetroxide for 2 h. At last, the fixed specimens were dehydrated in graded alcohols and propylene oxide, embedded in Epon 812, and cured for 48 h at 55 °C. The harvested specimens were cut into ultrathin sections (70–80 nM), and autolysosomes were observed by FEI Tecnai™ G2 Spirit BioTWIN transmission electron microscope at an accelerating voltage of 120 kV (FEI Company, Hillsboro, OR, USA).

### 4.15. Statistical Analysis

All data are given as means  ±  standard error (SE). Statistical analyses were performed using two-sample Student’s *t* tests and ANOVAs followed by an LSD post hoc test. *p* < 0.05 was the threshold of statistical significance. 

## Figures and Tables

**Table 1:** Primers for quantitative Real-time PCR.

Genes	Forward Primers(5′ to 3′)	Reverse Primers(5′ to 3′)
*MKP-5*	TTAGACGACAGGGTAGTAGT	GCTGGATGAGGGCATATA
*Bcl-2*	AGGATTGTGGCCTTCTTTGA	TCAGGTACTCAGTCATCCAC
*BAX*	GGCAACTTCAACTGGGGC	CCACCCTGGTCTTGGATCC
*PDX-1*	AGCAGTACTACGCGGCCAC	GAGCGGGGGCACTTCGT
*GLP-1R*	ATCGCTTCAGCCATCCTTG	GCCGTGCTATACATCCACT
*CHOP*	ACGGAAACAGAGTGGTCA	CGCTCGATTTCCTGCTTG
*GRP-78*	ACCAAGAAGTCTCAGATCTT	TTGTCTTCAGCTGTCACTCG
*IL-6*	TTCACAAGTCCGGAGAGGA	GAATTGCCATTGCACAACTC
*MCP-1*	CAGCCAGATGCAGTTAATGCC	AGCCGACTCATTGGGATCAT
*iNOS*	ATCTGCAGACACATACTTTA	GCCAGCGTACCGGATGAGC
*p22phox*	GCCATTGCCAGTGTGATCTA	AATGGGAGTCCACTGCTCAC
*NOX-4*	ACAACTGTTCCGGGCCTGAC	TCAACAAGCCACCCGAAACA
*GAPDH*	GCACCACCAACTGCTTAG	GCAGGGATGATGTTCTGG

## Abbreviations

Bax	Bcl-2 associated X protein;
Bcl-2	B-cell lymphoma-2;
CHOP	C/EBP homologous protein;
EIF2α	eukaryotic initiation factor 2α;
ERK	extracellular-regulated kinase;
ERS	endoplasmic reticulum stress;
FFA	free fatty acids;
GAPDH	glyceraldehyde-3-phosphate dehydrogenase;
Glut-2	glucose transporter-2;
GRP-78	glucose-regulated protein-78;
GSIS	glucose-stimulated insulin secretion test;
HFD	high-fat diet;
IL-6	interleukin-6;
iNOS	inducible nitric oxide synthase;
IRE1	inositol-requiring enzyme 1;
LC3	microtubule associated protein 1 light chain 3;
JNK	c-Jun amino-terminal kinase;
MAPK	mitogen-activated protein kinase;
MCP-1	monocyte chemotactic protein-1;
MKP-5	mitogen-activated protein kinase phosphatase-5;
PA	palmitic acid;
PARP-1	poly (ADP-ribose) polymerase-1;
PDX-1	pancreatic duodenal homeobox-1;
ROS	reactive oxygen species;
T2DM	type 2 diabetes mellitus;
TEM	transmission electron microscope
